# Virtual Reality-Based Rehabilitation Therapy

**DOI:** 10.33549/physiolres.935590

**Published:** 2025-08-01

**Authors:** Jacek LORKOWSKI, Monika RAULINAJTYS-GRZYBEK, Mieczysław POKORSKI

**Affiliations:** 1 Department of Management Accounting, SGH Warsaw School of Economics, Warsaw, Poland; 2 Center for Vision, Speech, and Signal Processing, University of Surrey, Guildford, United Kingdom; 3 Department of Management Accounting, SGH Warsaw School of Economics, Warsaw, Poland; 4 Institute of Health Sciences, Opole University, Opole, Poland; 5 Institute of Physical Education and Health, State University of Applied Sciences, Raciborz, Poland

**Keywords:** Advanced technology, Neuromuscular disorder, Rehabilitation therapy, Stroke, Virtual reality

## Abstract

This minireview describes recent advances in high-tech innovations on virtual reality (VR) aiding physical and cognitive recovery from neuromuscular disorders, notably useful for post-stroke rehabilitation. VR is a computer-generated technique that engulfs users in 3D multisensory interactive feedback. This technique creates simulations of realistic situations that can be manipulated by a user. It provides a spectrum of benefits in both physical and cognitive rehabilitation in the wake of neuromuscular episodes. VR engages and motivates patients to endure the unpleasant sequela of disease. Further, it enhances the acquisition of rehabilitative skills by caregivers and trains them in psychophysical health preservation. The benefits and user-friendliness of VR make it an increasingly welcome assistive neurological therapy tool. However, VR standardization, mechanisms, and, particularly, the long-term effects appear not to keep pace with its popularity and fast-progressing technical advances. Evidence-based studies on large groups of individuals are needed to settle these issues.

## Virtual Reality: Technique and Mechanisms

Virtual reality (VR) refers to computer-generated simulations immersing users in interactive, three-dimensional environments through multisensory visual, auditory, and tactile feedback [1,2]. Wearing head-mounted displays (HMD), such as the “Oculus Rift” (Oculus VR, Menlo Park, CA, USA) or “High-Tech Computer Vive” (HTC Corp., Taoyuan City, Taiwan), users perceive stereoscopic 3D visuals mimicking natural perception. These devices track head and body movements using accelerometers, gyroscopes, and external cameras, ensuring real-time synchronization between the user’s actions and virtual world updates [3,4]. VR systems’ engines make dynamic environments that adapt to users’ inputs [1,5]. A latency between movement and visual response must be below 20 ms to prevent motion sickness, which is achieved through high-refresh-rate displays (90–120 Hz) and advanced graphics processing units (GPU) [6,7]. Spatial audio algorithms enhance realism by simulating directional sound, for instance, footsteps echoing in a virtual hallway [8].

In health care, VR enables risk-free surgical training through hyper-realistic simulations, improving skill acquisition for medical professionals [5,9,10]. Therapists employ controlled virtual scenarios, like heights or crowded spaces, to treat phobias and post-traumatic stress disorder via gradual exposure [11,12]. A challenging issue for VR users is motion sickness caused by sensory conflicts between visual stimuli and the body’s vestibular system, although recent advancements in tracking precision reduce this issue [13,14]. The cost is a barrier since high-fidelity VR needs powerful hardware. The ongoing research strives to integrate the neural interface with photorealistic graphics to deepen immersion, making VR evolve from a niche to a mainstream medium [15–17].

## Virtual Reality in Stroke Treatment

Stroke is a devastating human pathology with uncertain recovery outcomes due to irreparable loss of neural plasticity and dysfunctional sensorimotor performance. Cerebral ischemia-reperfusion episodes, oxidative stress, proinflammatory sequela, and neuronal mitochondrial dysfunction are the pathological underpinnings of stroke episodes [18–20]. Preservation of a reasonable quality of life in post-stroke survivors is a formidable challenge. The research documents that VR therapy benefits in post-stroke upper limb mobilization. It improves the limb’s range of motion, dexterity, and grip strength, as assessed by the Box and Block and Action Research Arm tests [21], the effects being best when the therapy schemes last over 15 hours [22]. VR integration into conventional rehabilitation increases the Fugl-Meyer Assessment for Upper Extremity and Manual Function Test scores [23,24]. The electromyography-VR interface mitigates upper limb dysfunction by improving the neuroplasticity of muscle activation [25,26]. Combining VR therapy with non-invasive brain stimulation reduces spasticity and improves daily functionality, as assessed by the Modified Ashworth Scale and the Modified Barthel Index [27]. VR improves balance and mobility by increasing gait speed and stride length, as assessed by the Berg Balance Scale and Timed Up and Go test. The improvements persist for 12 weeks post-therapy [28,29]. Combining VR with functional gait exercise improves dynamic balance and postural stability better than VR alone [30,31]. VR therapy in stroke and general neurological rehabilitation is gaining increasingly widespread use. The following is the current internet and literature-retrieved list of major medical centers using it in various research and practice configurations:

- RENEURAL - in partnership with the Hillingdon Hospitals NHS Foundation Trust and Brunel University London, UK [32].
- University of Madeira, Funchal, Portugal, and Polytechnic University of Valencia, Spain [33].
- University of Messina, Italy [34].
- National University of Sciences and Technology, Islamabad, Pakistan, and universities in the Middle East [35].
- University of Michigan, Ann Arbor, MI, USA [36].
- McGill University, Montreal, Quebec, Canada [37].
- University of New South Wales, Sydney, Australia [38].

## Virtual Reality in Cognitive Impairment

Another area of VR application is cognitive rehabilitation. VR enhances working memory, language function, and visuospatial processing, particularly when combined with transcranial magnetic stimulation or direct current stimulation [39]. Improvements in self-confidence and self-reliance enhance psychosocial interactions. Increasing brain plasticity promotes post-stroke cognitive recovery [40–42]. Innovative technologies of extended reality (XR) and immersive VR yield further benefits. Systems such as Box and Block Test-Virtual Reality-Hand Tracking (BBT-VR-HT) and Mobility Device-Mixed Reality (MD-MR) provide remarkable clinical outcomes [43]. Gamified VR improves dexterity and grip strength in patients in the subacute phase of stroke, increasing motivation for exercise [44,45]. VR improves spatial navigational memory skills, affected by a stroke [46]. VR’s influence on patient quality of life and satisfaction cannot be underestimated. VR engages patients, which enhances its effectiveness and often makes it the treatment of choice for patients and rehabilitators alike.

## Limitations of Virtual Reality in Post-Stroke Rehabilitation

VR application requires careful consideration of patient-specific contraindications to ensure safety and efficacy. Severe cognitive impairment or dementia presents a significant barrier, as patients may have dysfunction of executive functions needed to run VR interfaces or interpret virtual stimuli [47]. Photosensitive epilepsy is an absolute contraindication due to the risk of life-threatening seizure induction from high-frequency light flashes or dynamic visual patterns inherent to VR environments, albeit no such events have yet been reported [48]. Caution is necessary when vestibular instability or severe dizziness is present, for these conditions may exacerbate cybersickness symptoms, including nausea and disorientation, as the unstable balance system struggles to reconcile conflicting sensory inputs [49,50]. Sensorimotor instability is a leading aftereffect of stroke. Aphasia hinders VR use due to impaired verbal communication that limits the patients’ ability to report discomfort or follow instructional cues [47]. Elevated intracranial pressure is a critical concern in using immersive VR that transiently increases cerebrospinal fluid dynamics, posing risks for individuals with uncontrolled hypertension [51]. During the hyperacute post-stroke phase (< 72 hours), VR interventions are contraindicated due to neural vulnerability in this critical recovery window [52,53]. Severe upper limb paresis, with the Medical Research Council (MRC) scale score less than 2, precludes effective interaction with VR controllers, which may require the introduction of patient-tailored input modalities [54]. Proliferative retinopathies and glaucoma require caution, for transient intraocular pressure fluctuations during VR may worsen optic nerve damage [55]. The contraindications outlined above show a need for pre-intervention screening and individualized risk-benefit assessment before using VR protocols.

## Virtual Reality Training Needs

Research shows that over 60 % of stroke caregivers lack knowledge about recovery milestones, best rehabilitation, or secondary stroke prevention, let alone innovative techniques such as VR, which could improve physical and mental functions [56,57]. Caregivers often feel unprepared to address post-stroke depression or cognitive decline. Over 80 % of caregivers are overwrought, of which about 7 % are heavily so [58–60]. They often lack practical clues for safely aiding patients through mobility transitions, adapting home environments, and implementing feeding protocols for those struggling with dysphagia [61,62]. Systemic barriers include poor coordination between healthcare and social services. Over 40 % of families of stroke-stricken patients lack information about available financial aid or respite care options, especially in resource-limited regions [63,64]

The EU CaregIVR project has been an innovative multinational university initiative oriented toward medically compromised patients and caregiver needs by combining modern technology with practical psychosocial support [65]. It aims to foster knowledge exchanges to tackle neuromuscular rehabilitation, not the least of which is training and psychological help for informal caregivers, often insufficiently taken care of by healthcare systems that focus mostly on patients. The emphasis is put on knowledge about aphasia, spatial anxiety, mobility disorders, and practical skills related to the patient’s nursing, and supporting rehabilitation while avoiding mistakes that could perpetuate abnormal movement patterns. The project highlights the VR potential to simulate the patient’s realities by offering immersive educational scenarios, like the visualization of stroke effects, simulation of daily challenges, or hands-on training in care techniques. Additionally, formal and administrative problems are discussed, including the barriers that make it difficult for caregivers to take advantage of available services. The VR implementation requires it to be tailored to different end-users in simple mobile forms, like smartphone applications, to achieve specific tasks performed by senior people who may not be skilled in running modern technologies. Gamification elements are needed to increase patient engagement and motivation. Example scenarios include simulations of the patient’s home environment or emergencies, like recognizing signs of stroke recurrence. Another important aspect is the creation of follow-up modules to verify and update patients’ skills and supervision.

## Conclusions

VR tools are of help in a knowledgeable, empathy-driven approach to the victims of stroke and other neuromusculoskeletal disorders. VR advantages concern both patients and caregivers. Importantly, the technology must be adapted to end-user needs by including psychological and administrative aspects in the tool design. Patients and caregivers alike benefit from VR therapy. VR is the epitome of how advanced technology may effectively aid patient management by combining technology with practical psychosocial support in a patient-tailored manner. Yet, there is a need to standardize VR therapy and its advancing variations and conduct larger clinical trials to assess the long-term effects. VR therapy is poised to refine neurological rehabilitation outcomes.
